# Hybrid Technologies Combining Solid-State Sensors and Paper/Fabric Fluidics for Wearable Analytical Devices

**DOI:** 10.3390/bios11090303

**Published:** 2021-08-28

**Authors:** Meritxell Rovira, César Fernández-Sánchez, Cecilia Jiménez-Jorquera

**Affiliations:** 1 Instituto de Microelectrónica de Barcelona (IMB-CNM), CSIC, Campus UAB, Bellaterra, 08193 Barcelona, Spain; meritxell.rovira@csic.es (M.R.); cesar.fernandez@csic.es (C.F.-S.); 2 CIBER de Bioingeniería, Biomateriales y Nanomedicina (CIBER-BBN), Jordi Girona 18-26, 08034 Barcelona, Spain

**Keywords:** wearables, paper microfluidics, fabric microfluidics, solid-state sensors, electrochemical (bio)sensor, clinical analysis

## Abstract

The development of diagnostic tools for measuring a wide spectrum of target analytes, from biomarkers to other biochemical parameters in biological fluids, has experienced a significant growth in the last decades, with a good number of such tools entering the market. Recently, a clear focus has been put on miniaturized wearable devices, which offer powerful capabilities for real-time and continuous analysis of biofluids, mainly sweat, and can be used in athletics, consumer wellness, military, and healthcare applications. Sweat is an attractive biofluid in which different biomarkers could be noninvasively measured to provide rapid information about the physical state of an individual. Wearable devices reported so far often provide discrete (single) measurements of the target analytes, most of them in the form of a yes/no qualitative response. However, quantitative biomarker analysis over certain periods of time is highly demanded for many applications such as the practice of sports or the precise control of the patient status in hospital settings. For this, a feasible combination of fluidic elements and sensor architectures has been sought. In this regard, this paper shows a concise overview of analytical tools based on the use of capillary-driven fluidics taking place on paper or fabric devices integrated with solid-state sensors fabricated by thick film technologies. The main advantages and limitations of the current technologies are pointed out together with the progress towards the development of functional devices. Those approaches reported in the last decade are examined in detail.

## 1. Introduction

In recent years, efficient, simple, and low-cost diagnostic tools for decentralized rapid analysis of biomarkers at home, the point-of-care (POC), the point-of-need, or over-the-counter (OTC) have been highly demanded. Reports show that the diagnostic market size will be doubled within the next five years, this being recently boosted by the COVID-19 pandemic. The pace at which these devices are developed has been possible with the advancements achieved in materials, electronics, and manufacturing techniques. Two main groups can be identified regarding the device ability to provide a qualitative (yes/no) or quantitative result. While the former have shown widespread application mainly due to the implementation of easy and very cost-effective instrument-free lateral-flow strip devices [1], the development of quantitative approaches has been more challenging considering the strict requirements in sensitivity, specificity, and precision that should be fulfilled.

Quantitative POC devices are ideally compact and comprise miniaturized sensor components integrated in a device that allows for the sample uptake, processing, and analysis in an automatic or semi-automatic fashion. This can be done with fluidic approaches and electronic or photonic components for sensor interrogation and signal recording and interpretation.

The different elements of these POC devices are detailed and examined in the following sections.

### 1.1. Microfluidic Elements

Microfluidic elements are required in POC devices considering that the sample volumes usually available in clinical diagnosis are in the scale of microliters or below. Microchannels showing different geometries are included together with flow cells for sensor implementation, as well as reagent reservoirs and actuating components such as pumps and valves [2] for driving the sample and required reagents towards the device sensing area. The integration of pumps and valves is a key issue in microfluidics, and many devices reported so far rely on external components that make them of limited use for decentralized analyses. Few of them have gone into the market being the iSTAT system commercialized by Abbot the most representative one [3].

Among the different pumping strategies that have been assessed in the field of microfluidics, those that rely on flow by capillary action can suit a great variety of applications that make use of small liquid volumes, and the overall sample analysis requires very few steps. The inherent porosity of paper and other related materials enables the liquid to flow through them by capillary action without the need for an external pumping source [4]. In this context, the concept of microfluidic paper-based analysis devices (μPADs) has been created in recent years and provides a feasible low-cost alternative to traditional laboratory tests for the diagnosis of many common diseases and disorders [5]. Emanuel Elizalde et al. investigated the capillary inhibition in paper-like substrates to better understand fluid transport in terms of the macroscopic geometry of the flow domain [6]. This study concluded that capillary driven flow is determined by two main factors, that is the physicochemical properties of the interfaces involved and the geometry of the flow. Besides, paper has advantages over other materials, including low cost, simple production, good chemical stability, flexibility, biocompatibility, lightweight, and hydrophilicity. In addition, its surface can be easily modified, cut, folded, and/or stacked. This material has also been the basis of the well-known lateral flow devices with the most popular example being the glucose meter [7] and the very recently developed COVID-19 POC devices [8].

Regarding textiles, gaps between fibers in the fabric provide capillary channels for liquids to wick along threads without the need of an external pump. Their capillarity properties can be modified with standardized methods commonly used in paper microfluidics such as wax printing, photolithography, or cutting [9,10]. Besides, to get smart clothes, new composite and nanomaterials have been found to be promising candidates to obtain improved performance of textile based wearable devices [11].

### 1.2. Sensor Types

The integration of the sensing part on POC devices is diverse and has generated a huge number of reviews. Detection is an important challenge in the context of POCs, considering that the sensing equipment may be miniaturized, portable, easy to use, and non-expensive. Although a large variety of methods have been used for the detection of analytes, colorimetric sensing is surely the most prevalent [12,13]. Colorimetric chemicals can be easily integrated in paper and textile substrates and results can be visually evaluated under the naked eye (yes/no answer) and may also provide semiquantitative results by using a calibration chart, a handheld reader, or the camera of a smartphone. An interesting paper describing the measurement of pH and lactate on a textile substrate shows the simplicity of this approach for semiquantitative analysis [14]. However, they cannot provide real-time, continuous, and quantitative information of the target analytes being analyzed.

For this, it has been shown that chemical sensors mostly based on electrochemical transduction modes enable analyte continuous monitoring, providing rapid and reliable quantitative results, and can be adapted for different healthcare applications. However, integration of both paper and fabric-based microfluidics and chemical sensor devices has been challenging, and different innovative designs have been reported so far, as will be explained in the following sections. Electrochemical techniques offer high sensitivity, low detection limits, possibility of enhancing selectivity by applying different potential of modifying the electrodes, and inexpensive. In order to facilitate the integration of electrochemical sensors in POC structures, miniaturized solid-state sensors should be used. Their size can be scaled to micro- and nano-level, they can be easily implemented in fluidic systems, they require low amounts of sample, electrodes can be shaped as required and they can be integrated into almost any type of platform or substrate. The technologies used for solid state electrochemical sensors are generally thin film deposition (e.g., photolithography, evaporation and chemical vapor deposition of metal layers) and thick film deposition (e.g., inkjet printing and screen printing). Most of the works described in this review use the later technique. Screen printing (SP) is a popular electrode fabrication technique as it uses inexpensive equipment, can be automatized, can be scaled up for mass production, and it is user-friendly [15]. It uses screens or meshes to pattern printed electrodes on a wide range of substrate materials. Conductive ink is spread onto previously designed screens or meshes with the desired electrode geometries placed on the substrate and they are thermally cured at 60–90 °C for several minutes. Screen-printed electrodes (SPEs) are not only easy to fabricate but also can be simply modified by adding reagents and particles to the inks. However, the electrochemical properties of SPEs are unfortunately not as good as traditional metallic electrodes in terms of electron transfer kinetics and electrode resistance. Inkjet printing is now becoming a great alternative to SP due to the possibility to fabricate more miniaturized patterns and integrate printed circuits in the same substrate [16]. As an example, an organic electrochemical transistor (OET) inkjet printed on a flexible polyimide foil (PI) and applied to sweat measurement is described in reference [16,17].

All these techniques use substrates for wearable devices that are compatible with the sensor fabrication technique requirements (i.e., temperature, mechanical robustness, etc.). The most usual materials are polymethyl methacrylate (PMMA), with polyimide (PI) belonging to the brand Kapton being one of the most popular, and polyethylene terephthalate (PET).

Regarding thin film deposition techniques for sensor fabrication, the most representative are those for fabrication of ISFETs with silicon substrates and flexible substrates [18,19,20].

**Figure 1:**
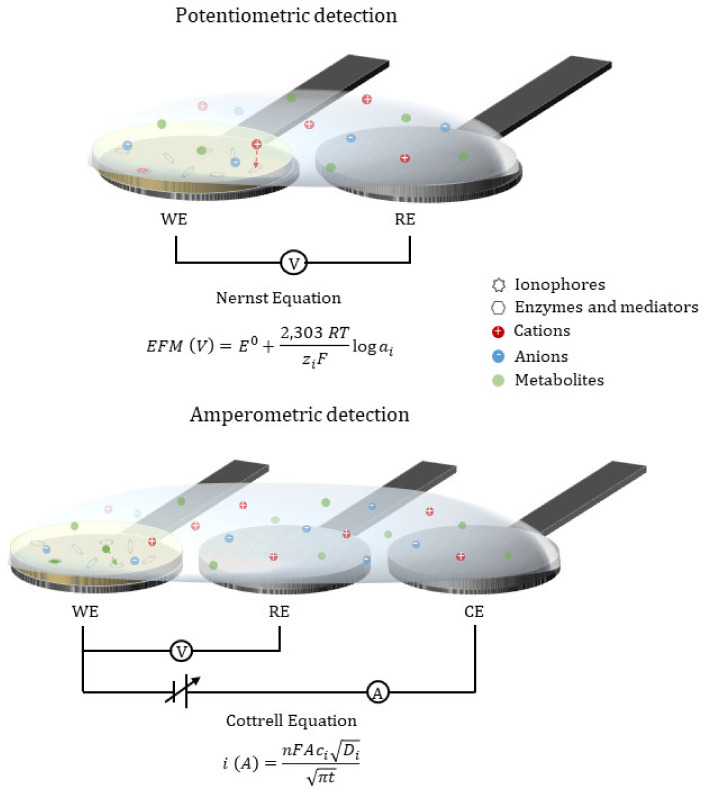
Potentiometric and amperometric sensors schemes and mechanisms of detection.

Among electrochemical detection, potentiometric ion selective electrodes are usually employed in POCs to quantify analytes that are not redox active (i.e., ions and pH) [21]. The change in voltage signal between the working and the reference electrode at close to zero-current conditions when the ionophore binds to the analyte is proportional to the activity of the analyte in the sample. This concentration dependence is described by the Nernst equation and confers to potentiometry the capability to quantify. In Figure 1, the schematic of a potentiometric sensor is shown.

In order to determine species that are redox active, amperometric and voltamperometric techniques can be used [21]. These are the most usual techniques for the detection of biomarkers like glucose and lactate as the corresponding enzymes catalyze redox reactions. These amperometric sensors consist of a biocatalyzer, an electronic mediator, and an electrochemical transductor. The intensity recorded when the redox active specie is oxidized/reduced could be related to the amount of analyte by Faraday’s law and more specifically to the Cottrell equation when the chronoamperometry technique is used—a potential is fixed between the working and reference electrode—and the reaction is controlled by diffusion (Figure 1).

### 1.3. Applications in Healthcare

**Figure 2:**
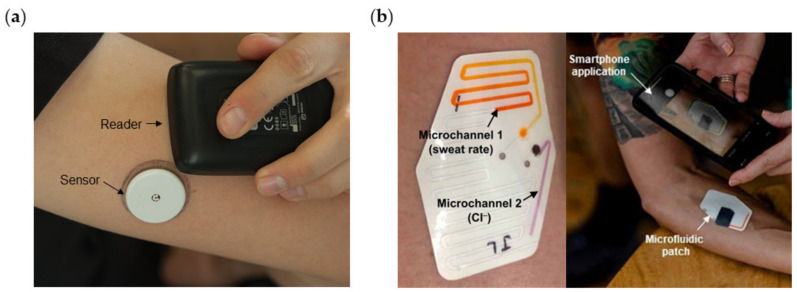
(**a**) Picture of FreeStyle Libre system from Abbot, consisting of a sensor worn on the arm and a handheld reader. (**b**) Picture of Gx Sweat Patch from Epicore Biosystems worn on the arm and read with a smartphone. Reprinted with permission from [25] for panel (**a**). Reprinted with permission from [24], Copyright 2020 American Association for the Advancement of Science for panel (**b**).

Many resources have been recently invested in order to improve people’s healthcare by prevention and continuous diagnosis. Pharmaceutical companies such as Roche or Janssen are developing their own wearable devices for diagnosis and better understanding of diseases such as Parkinson’s, Huntington’s, or heart-related injuries. Furthermore, an increase in the number of diabetes cases has pushed medical tech companies into wearables business and one of the most popular diabetes monitoring devices is the FreeStyle Libre by Abbott [22]. It is a 14-day system that invasively and continuously monitors glucose levels. As shown in Figure 2a, it is worn on the back of the upper arm and results are transmitted to a reader or smartphone.

One of the last revolutions in the area of health monitoring and POC devices is in sports. Companies such as Fitbit, Garmin, and Apple have developed new platforms that enable the monitoring of consumer’s own basic health (e.g., physical activity, heart rate, and sleep stages) in their daily lives [23]. However, reliable monitoring of (bio)chemical parameters has not been achieved yet and important investments have been made in this direction.

One of the major challenges of this decade is the continuous, noninvasive monitoring of biomarkers in sweat by means of wearable devices. The analysis of several biomarkers provides useful information about the physiological state of a person. Sweat is composed mainly of water (99%), but it also contains electrolytes such as sodium, chloride, and potassium, and metabolites like urea, pyruvate, and lactate. Sweating leads to the loss of water and electrolytes due to thermoregulatory action of sweat during exercise. The measurement of these electrolytes could provide information of the individual health status. Regarding to commercial wearable devices that measure sweat biomarkers, only the Gx Sweat Patch from Epicore Biosystems in partnership with Gatorade and Pepsico is capable of continuously measure biochemical markers in sweat. The device components are shown in Figure 2b. It provides a colorimetric feedback measurement of sweat rate, fluid loss, and sodium loss in real time [24].

The development of wearable devices based on electrochemical sensors for in situ sweat biomarker detection was first published in 2010 by the group of Diamond from Dublin University that developed a Na ion selective electrode (ISE) wearable device and was applied to compare the change in concentration of this target species during exercise done by patients suffering from Cystic Fibrosis (CF) and healthy patients [26]. Since then, several groups have been developing different wearable devices for monitoring biomarkers (pH, sodium, chloride, potassium, and lactate) in sweat. In most of them, no microfluidic components are applied and just absorbable surface in direct contact with the sensing element is used for collecting sweat and then being measured.

Some of the wearable devices described in the literature integrate the sensor in the same paper or fabric. This can be modified with conducting organic (polyaniline, polypyrrole, polytiophene, etc.) or inorganic (metal, metal oxide, etc.) materials to be conductive enough to perform the transduction of the chemical signal to the electronic signal. Techniques such as dip coating, printing, or sputtering are used to modify paper and fabric fibers [27,28]. For instance, a paper-based glucose sensor integrated into a standard Band-Aid adhesive patch is described in [29]. It used a 3D paper-based fuel cell configuration that enabled glucose monitoring without external power supply. On the other hand, it has been recently reported a wearable origami-like paper-based platform for sulfur mustard detection [30]. A wax pattern was defined onto a filter paper sheet and the three-electrode system was printed with conductive inks. They used different layers of paper to pre-load reagents needed for the enzymatic bioassay separately. In a similar approach, a paper-based wearable platform for continuous and real-time analysis of lactate and glucose in sweat was lately developed [31]. At first, fluidic channels and reservoirs were defined with hydrophobic wax in a paper sheet. Later, electrodes were screen-printed and, at last, paper was folded into a multi-layer structure that enabled a vertical flow of sweat.

The group of Wang was early in describing a SP biosensor demonstrating the potentialities of sensor integration in clothing for sport and defense applications [32]. A more simple approach to build electrochemical sensors for pH, K, and NH_4_ using cotton yarns is described by Andrade group in [33]. Yarns were turned into electrical conductors through a carbon nanotube ink and covered with a polymeric membrane to obtain ion-selective electrodes. Through the placement of these sensors in a band-aid, this approach could be easily implemented in a wearable device. Another interesting approach made use of a single cotton yarn as gate of an organic electrochemical transistor (OET) to detect adrenaline for hydration and stress human monitoring [34].

However, to date, these devices integrating the sensing element in paper and fabrics are far to provide reliable quantitative results. In addition, they usually lose microfluidic capabilities of the substrate.

Generally, when microfluidic components are considered, PDMS has been the material of choice for most of the microfluidic devices developed in research laboratories. PDMS has been undoubtedly key for the development of microfluidics due to the ease of producing structures like microchannels of different geometries as well as other fluidic components and of integrating sensor devices allowing rapid prototyping and proof-of-concept demonstrations [35]. However, this material can show drawbacks depending on the channel size like the formation of bubbles (mild wettability), long time of response, and poor chemical compatibility. PMMA has also been used in microfluidic structures. An example is that described by the group of Diamond for colorimetric pH detection in sweat [36].

Other materials like PET, SU-8, and COC/COP have also been considered to different extent [17,20,37]. As already pointed out, paper is an alternative material for microfluidics in point-of-care devices in general and wearable devices in particular due to its capacity of driving the sweat to the sensor areas while working, but also to collect the sample and accumulate it after carrying out measurements in continuous applications. The use of paper channels ensures obtaining fresh sweat from the skin surface that is continuously driven through the microfluidic channel by capillary action towards the sensors. Besides, fabrics are materials equivalent to paper as they also use capillary forces to drive the samples over the sensors and they show the particularity of producing channels themselves without the need of patterning [9,10,38,39,40,41]. Devices that integrate paper or fabric materials meet the “ASSURED” criteria (affordable, sensitive, specific, user-friendly, rapid and robust, equipment-free, and deliverable to users) set by the World Health Organization (WHO) for diagnostic tests [42].

This review describes all reported works focused on paper- and fabric-based microfluidic devices that integrate solid-state sensors applied to wearable systems for sweat analysis. The hybridization of the sensor component layer with the microfluidic component enables the continuous monitoring of the target analytes, using low-powered simple instrumentation that can be supplied wirelessly. However, the integration of both parts is one of the major difficulties in wearable devices. Its potential has not been exploited yet because of the technological challenges still to be worked out. The potential of such type of devices is foreseen and that is why it deserves being summarized in this short review.

## 2. Paper-Based Microfluidics for Wearable Devices

Among the many strategies toward capillary-driven microfluidics, paper is considered the most attractive and promising substrate material. It has unique advantages such as its extremely low cost and its great mechanical properties (flexible, light, and thin). Its porous structure provides capillary transport, capacity of storage of reagents, air permeability useful to avoid air bubble formation, filtration, and high surface to volume ratio that results in short time for the analysis [43]. Due to its inherent biocompatibility, biodegradability, sustainability, and disposability, as well as chemical and biological inertness, it is particularly important for the immobilization of bioreagents, and it also fits into the so-called circular economy model.

Among the different paper-like materials available in the market, filter papers of different grades show special features, in terms of physicochemical properties in terms porosity or overall structure, which make them especially suited for microfluidic devices. Traditional paper is made of cellulose but porous flexible substrates made of nitrocellulose (NC), bio-source photonic crystals, and flexible softshell particles have also been considered as paper materials [44].

The first example of a paper microfluidic structure was described in 1937 by Yagoda Herman [45], who patterned hydrophilic spot tests on a paper substrate using paraffin. Using the same technique, in 1949, Müller et al. reported a paraffin-patterned paper microfluidic channel [46] for improving the performance of a paper chromatographic device. However, it was not until the first decade of the 21st century that paper microfluidics drew the attention of the scientific community [47]. In 2007, Whitesides’ group at Harvard University introduced the first paper-based device for the detection of glucose, fabricated by a lithographic method and pioneered the term “paper microfluidics” [48]. Since then, a large variety of fabrication techniques for producing paper-based microfluidic devices have been developed.

Paper is patterned to construct the required microfluidic architecture and two main approaches have been applied in this regard: (1) two-dimensional shaping/cutting of paper and (2) patterning hydrophilic/hydrophobic areas on paper. The former mainly comprises cutting the paper with a cutter plotter capable of shaping paper into previously customized designed or with a CO_2_ laser cutting apparatus. The latter is based on creating a hydrophilic/hydrophobic contrast by one of the following approaches: (a) Physical blocking of the paper pore structure. (b) Physical deposition of a hydrophobic agent on the paper surfaces. (c) Chemical modification of the paper surface.

Regarding the implementation of a detection approach into a wearable device, some features should be considered. This should be intuitive to operate but also portable and inexpensive, fulfilling the requirements for in field analysis. We can distinguish three different approaches for paper-based analysis: the naked eye, digital colorimetry, by using a smartphone for example that can read the color and intensity of a spot and instrumental analysis in which the signal is recorded by an instrument [49].

Colorimetric detection is the most popular one in paper-microfluidic devices, as paper offers a bright, high-contrast, and colorless background for color change readings. Numerous paper-based assays, such as the new COVID-19 antigen test and the pregnancy test, are simply interpreted under the naked eye and provide with a yes/no answer for qualitative analysis. Digital colorimetry based on the measurement of color intensity or pixel counting has also been of widespread use mainly because the only instrument that is required is a digital camera, which we find in every smartphone or portable device. However, semiquantitative analysis can be carried out using these approaches.

Instrumental analysis is necessary for quantitative biomarker analysis. Among the different options available, electrochemical detection has been the most widely used. Electrochemical paper-based analytical devices combine the inherent advantages of electrochemical detection, such as high sensitivity and low detection limits (LODs), affordability and the possibility of enhancing selectivity and sensitivity by applying different electrochemical techniques or using electrochemical cells made of different materials and showing specific configurations, with those of paper, already mentioned above [50].

Table 1 summarizes the main characteristics of some wearable devices that combine electrochemical analysis and paper-based microfluidics.

The first work showing the feasible integration of a microfluidic structure together with a solid-state sensor was reported by Heikenfeld’s group [51]. He developed a flexible patch containing a Na ISE and a paper microfluidic structure for continuous measuring this ion in sweat during exercise practice. One of the most relevant features of this patch was being battery-free and communicating wirelessly to an android smartphone. It used a radio-frequency inductively powered (RFID) chip in order to obtain the power for sensors reading. Unfortunately, due to the limitations of the analogue digital converter on the RFID, the Na sensor response was lower than the expected one. It also exhibited a slight drift (3–5 mV) due to the instability of the Ag/AgCl reference electrode. Its simple microfluidics design enabled low response times (30 s), but it was unable to be used for long-time measurements during exercise performance.

**Figure 3:**
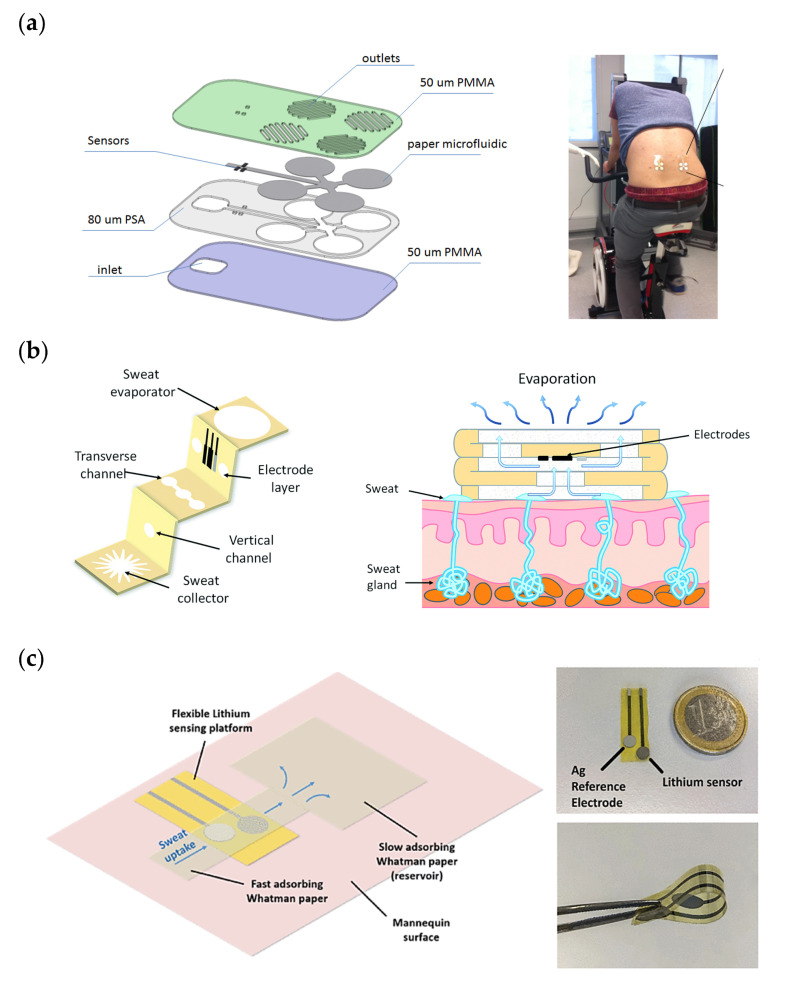
(**a**) Assembly of the different layers of the multi-sensing patch for sodium, pH, and lactate monitoring and picture of the device on-body. (**b**) Schematic of the three-dimensional paper-based device for glucose monitoring, including the different parts and its application on the human skin. (**c**) Photographs of the lithium-sensing layer and schematics of the wearable system including paper-microfluidics. Reprinted with permission from [52] and Copyright 2016 MDPI for panel (**a**). Reprinted with permission from [55] and Copyright 2019 The Royal Society for panel (**b**). Reprinted with permission from [58] and Copyright 2021 IEEE for panel (**c**).

Following this work, a flexible microfluidic platform for simultaneously and selectively measuring lactate, pH, and sodium in sweat by integrating amperometric and potentiometric detection approaches (Figure 3a) [52] was reported. Sensors consist of arrays of flexible 50 µm diameter flexible microneedles implemented in microfluidic paper channels. The sensor analytical performance showed that they stabilized very rapidly, within 10 s of starting the measurements, this being a relevant feature for continuous monitoring of the target biomarkers. Long-term in vitro studies carried out with this device showed stability and repeatability for over 8, 5, and 4 months for the pH, sodium, and lactate sensors, respectively. Sample flow control in the device is achieved by using papers of different grades. On-body tests were carried out on six healthy males during cycle ergometry and treadmill running, obtaining reliable results for all the sensors that were comparable to those recorded in previously reported works.

More recently, a wearable patch for lactate monitoring in sweat that make use of an osmotic-capillary microfluidic pumping approach for controlling the flow [53], was developed. This was possible by incorporating an hydrogel film at the sweat collector area of the device, which, due to the high content of ions, had the effect of pumping by osmotic force and enabled continuous extraction of fluid from the skin surface [54] while the paper-based channel drove the fluid to the sensor by capillary wicking. This paper only reported in vitro tests, where the lactate sensor showed a linear response in the relevant physiological range of 5 mM to 20 mM.

In a more complex approach, a three-dimensional paper-based microfluidic structure was used in the production of a patch for glucose detection in sweat [55]. A patterned paper was folded into an origami structure with five equal layers in order to form the 3D flow channel. This microfluidics design included five layers: sweat collector, vertical channel, transverse channel, electrode layer, and sweat evaporator (Figure 3b). This configuration enabled the separation of the electrodes from skin, fast response, and continuous evaporation leading to continuous renewal of the sample over the electrodes. The 3D device was tested using a flow injection system that simulate the flow of sweat. The results displayed a good repeatability and linearity in glucose concentration ranging from 0 to 1.9 mM covering the range of glucose physiological concentrations in human sweat (range: 0.25–1.5 mM). However, the sensitivity of the sensor was lower than in the test without microfluidics due to the absence of stirring. On-body tests were performed placing the system on the forearm of three subjects. The results showed that glucose concentration decreased gradually during exercise (from 1.5 mM to 0.4 mM) and that there was a significant variability between the three subjects. A second generation of this device was developed that integrated a potassium electrochemical sensor. The new version of the device was tested for real-time monitoring of this target species [56]. It showed a small size, and the results reported a quasi-Nernstian response, 5 s response time, and a stable performance within 1 week. An interesting feature that was studied in this work is the patterning of the sweat collection area. Three designs were tested: circular, radial, and snowflake-like structures. The circular pattern covered the maximum of grids among the three of them, but it required the largest sweat volume (20 µL) to fill it and so a long time for sweat to reach the electrodes zone was necessary before a signal could be measured. In the radial design the area in contact with the skin was reduced, and so the volume required for filling it. The specific structure made the collector area to be more efficient for sweat collection and transport. The snowflake-like design showed more branches of smaller width, reducing the hydrophilic area contacting the skin even further. Therefore, the snowflake-like pattern showed the most efficient sweat collection and the fastest response. Real-time analysis was performed on volunteers during cycling with the device attached to the forearm. Results were consistent with the previous literature reporting potassium measurements in sweat. However, the mechanism of sweat potassium loss is still unclear.

The hybridization of paper microfluidics and sensors defined on a different substrate can also be used for non-chemical based systems. An example of this is reported in [57] where a sensor for measuring sweat rate is described, which consisted of printed interdigitated electrodes and a serpentine shaped paper-based microfluidic channel. By calculating the fluid volume per serpentine line, the time values at which the fluid crosses the fingers of the interdigitated electrodes, and by measuring the admittance between the two interdigitated electrodes, sweat rate could be monitored. The device could hold sweat volumes up to 82 µL in 30 min filling time and could be placed on various locations of the body for extended periods. This device was validated in vitro.

In a recent paper, a wearable electrochemical sensing platform was developed to monitor lithium in sweat for therapeutic drug monitoring of people suffering from bipolar disorder [58]. A potentiometric lithium ion-selective electrode (ISE) was integrated on a flexible polyimide (PI) substrate. The microfluidics design was simple but very effective. A fast-absorbing paper was implemented on the sample collection area and sensing regions to ensure the effective renewal of sweat on the electrode area. Quasi-Nernstian behavior of the sensor was proved with artificial sweat samples, providing good analytical capability for the target application. Neither on-body tests nor tests simulating typical sweat flow rate (1.5 µL cm^−2^ min^−1^)) were reported, so the response time of the sensor and the maximum operating time of the platform are unknown. This device has lately completed with more sensors and cotton has been used instead paper as microfluidic element [59].

## 3. Fabric-Based Microfluidics for Wearable Devices

As a result of the continuous efforts put to find new substrate materials for microfluidic platforms, simple and inexpensive fabric-based microfluidics have emerged. This research arrived soon after the paper-based microfluidic analytical technology in 2010 [9,10]. As well as in paper microfluidics, gaps between fibers in the fabric provide capillary channels for liquids to wick along threads without the need of an external pump. Their capillarity properties can be modified with standardized methods commonly used in paper-microfluidics such as wax printing, photolithography, or cutting.

Fabric-based microfluidic technologies are affordable, flexible, robust, user-friendly, quick, and scalable. They can be fabricated in 2D or 3D configurations to transport fluids depending on the application [60]. Both paper and fabric can be combined to take advantage of the special features of both materials. Thread can transport liquids with no need of patterned barriers, while paper can facilitate the immobilization and in turn integration of enzymes and reagents [9,61]. In 2013, Pan and coworkers proposed a novel interfacial microfluidic transport principle using micropatterned superhydrophobic textiles [62]. It consisted of stitching patterns of hydrophilic cotton yarns on a superhydrophobic cotton textile treated with fluoropolymer microparticles. The system was developed aiming at achieving a continuous and controlled sample flow but lacked a biochemical testing application.

Just like in paper microfluidic devices, thread is also suitable for fabricating microfluidic electroanalytical systems. There are many different types of fibers that can be used for the construction of fluidic channels, which can be combined with electrodes for quantitative analyses. Among them, rayon, cotton, nylon, polyester, wool, or silk threads could be used that differ on the wicking rate. If the application requires fast-absorption areas, fibers with high wicking rates may be used. They can also be treated with plasma to increase their wicking rate [63].

**Figure 4:**
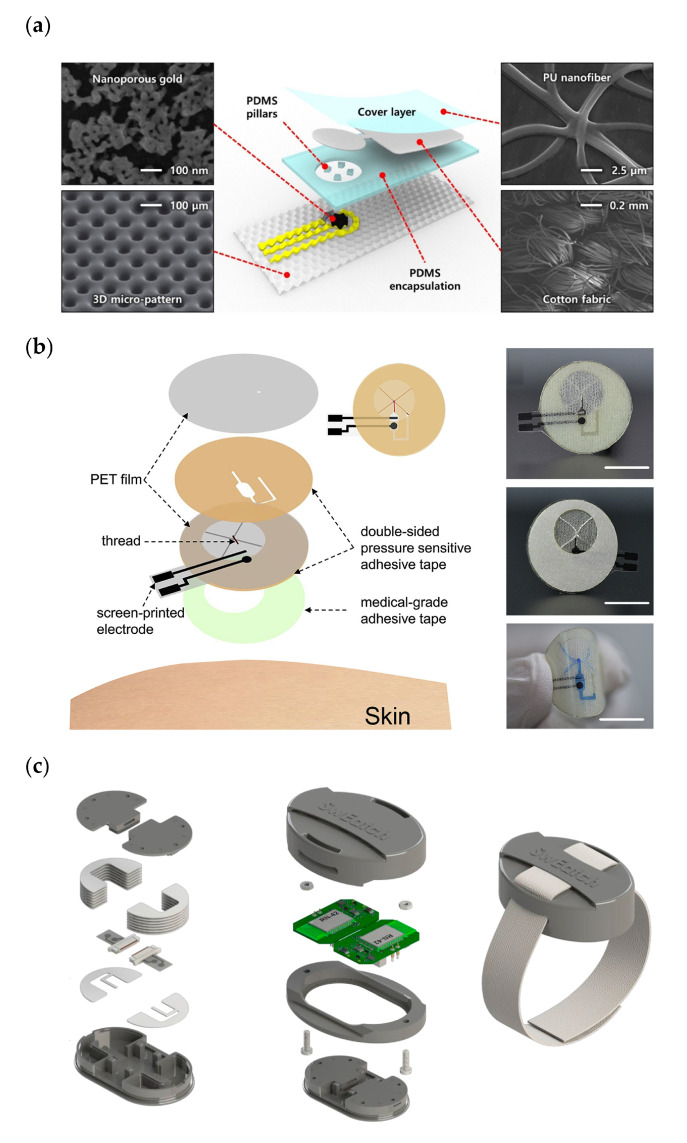
(**a**) Pictures of the glucose sensing patch on the forearm, demonstrating its conformality under compression and stretching, and schematic of the different layers conforming the device showing their SEM images. (**b**) Schematic showing the different components of the patch for sodium monitoring, and photographs of the device on the arm and forehead of an individual (threads colored with blue dye). (**c**) Schematics of the different layers of the microfluidic unit, of the platform body and view of the fully enclosed 3D printed SwEatch platform. Reprinted with permission from [64] and Copyright 2019 American Chemical Society for panel (**a**). Adapted with permission from [65] and Copyright 2020 Elsevier for panel (**b**). Adapted with permission from [66] and Copyright 2019 Elsevier for panel (**c**).

Few reported applications are focused on the hybridization of textile-microfluidics and solid-state sensors [64,65,66]. C. W. Bae et al. developed a fully stretchable sweat sensing patch for the detection of glucose with a non-enzymatic nanoporous gold (NPG)-based sensor and a textile microfluidic component (Figure 4a) [64]. Tests with different dyed water solutions showed filling times of ~16 min and good renewal of the solution. Sensitivities of 57 µA cm^−2^ mM ^−1^ were achieved without any hysteresis effect. A drawback of the system was that when sample volume was not enough, the sensor generated a false signal, thus being a serious obstacle continuous monitoring. To tackle it, the number of inlets in the sample collector area and the overall collector size were adapted considering the absorption rate of the fabric and the channel width.

Recently, a wearable device for the detection of sodium in sweat was reported [65] that consisted of fabric microfluidics and screen-printed electrodes implemented on PET films (Figure 4b). A patterned double-sided PSA was sandwiched between two PET films, forming a channel of 280 µm height in which a thread was placed. It required 8–11 min to fill the detection reservoir with just one inlet, this being a rather long time for continuous monitoring. Long-term stability tests resulted in a low drift (below 1 mV after 1000 s). On body Na monitoring provided concentrations in the normal physiological range.

Diamond’s group recently developed a watch type platform for monitoring Na and K in sweat [66]. They had previously reported the system called “SwEatch” only for monitoring sodium ions [67]. The microfluidics was based on two threads that draw the sweat from the skin to both Na and K sensors (Figure 4c). On-body trials showed sharp increases in the signal for both Na and K taking place when sweat reached the electrodes after 8 min. The device detected a lower Na concentration than expected, probably due to sweating rate, skin temperature, or electrolyte reabsorption.

The main characteristics of the previously described are summarized in Table 1.

## 4. Conclusions and Outlook

The research status and characteristics of paper- and fabric-based fluidics integrated with solid-state sensors was reviewed. All the works reported so far included sensors mainly fabricated by screen-printing techniques. One of the main drawbacks of this type of sensors is that they require large sample volumes, and the miniaturization capacity of microfluidics cannot be fully exploited. However, it was shown that all the reported approaches show great versatility and conformability (2D and 3D structures), their properties can be modified to control the sample flow, they are cost-effective and flexible to be incorporated into wearables, they do not require pumps or valves, and they allow continuous measurement. Having the sensing layer and the microfluidics in different substrates enables the integration of compact and simple electronics.

Few works test their devices on-body, which shows the difficulty of the final integration and stability of the measurements in real conditions. The ones assessed in a real scenario show promising results that successfully compared with previously reported information, rather than contrasting them with standard methods. Further work should be done in order to improve the sampling of biofluids and to be able to fully validate the devices.

Comparing the microfluidic characteristics of paper and fabric, it is clear that fabric-microfluidic devices have longer time responses than paper-based ones due to more limited wettability of the applied fabric. Fabric also displays a wide variety of interfiber gap sizes, resulting in a worse reproducibility than paper. On the other hand, thread has great tensile strength, flexibility, ability to easily form 3D structures and simplicity of being combined into wearable materials. Therefore, the optimal selection of an adequate material platform relies on the requirements of the application.

The current proof-of-concept demonstrations show a great opportunity for simplifying basic health monitoring, enabling self-diagnosis without needing complex instrumentation. However, the embedding of such devices with wearable electronics for direct sample collection and continuous monitoring deserves further investigation. Implementing microfabricated sensors in these wearable systems would offer clear advantages in terms of miniaturization, fabrication, reproducibility, degree of integration of not only sensor and microfluidic components, but also all the required electronics. It is still in its early stages, as it requires progress in innovation, knowledge and skills in fabrication techniques and detection methods to enable these platforms to reach maturity.

## Figures and Tables

**Table 1:** Main characteristics of the reported devices integrating paper and fabric microfluidics and electrochemical sensors on flexible substrates.

Integration of Substrate, Sensors and Microfluidics	Detection Technique	Sensor Technique/Type	Marker	Response Time *	Sensitivity (Linear Range)	Ref.
*Paper microfluidics*						
Paper microfluidics + ISE and RE on flexible Kapton substrate	Pot	Pd and Ag electrodeposited on patterned Cu electrodes for WE and RE	Sodium	30 s	0.3 mV/mM (10–90 mM)	[51]
PMMA layers and paper microfluidics + 6 electrodes placed inside a paper channel	Pot/Amp	Pt and Ag flexible microneedles for 3 WE, 2 RE and CE; pH IrOx membrane; Na WE coated with PEDOT; lactate LOx in BSA/PU + SPEES/PES	pH/ Sodium/ Lactate	10 s	pH: 71.9 mV/dec Na: 56 mV/dec	[52]
A hydrogel and paper microfluidics + WE, RE and CE on PI substrate	Amp	SP WE with Prussian Blue/carbon ink, RE with Ag/AgCl ink and CE with carbon ink; LOx and Nafion drop-casted in WE	Lactate	16–20 min	0.03 µA/(mM·mm^2^) (5–20 mM) LOD: 6 mM	[53]
3D wax-printed paper microfluidics + WE, RE and CE on PET substrate	Amp	SP WE and CE with Prussian Blue/graphite ink and RE with Ag/AgCl ink; GOx drop-casted in WE	Glucose	-	35.7 µA/(mM·cm^2^) (0–1.9 mM) LOD: 5 µM	[55]
3D wax-printed paper microfluidics + WE and RE on PET substrate	Pot	SP WE and RE with Ag/AgCl and carbon inks; WE coated with PEDOT:PSS	Potassium	5 s	61.8 mV/dec (1–32 mM)	[56]
Paper microfluidics + electrode on PI film	Imp	SP electrode with carbon ink	Sweat rate	30 min	-	[57]
Paper microfluidics + ISE and RE on a flexible PI substrate	Pot	WE (ISE) and RE by photolithography patterning	Lithium	-	56.8 mV/dec (2 mM–1 M) LOD: 1.7 mM	[58]
*Fabric microfluidics*						
Cotton fabric and PU nanofiber cover + WE, RE and CE on flexible PDMS substrate	Amp	Nanoporous Au WE and CE vacuum-deposited and Ag/AgCl RE	Glucose	16.15 min	57.6 μA/(mM·cm^2^)	[64]
Thread + ISE and RE on PET film	Pot	SP ISE and RE with graphite and Ag/AgCl inks resp.; ISE and RE coated with PEDOT and PVB resp.	Sodium	8–10 min	56.7 mV/dec	[65]
3D printed platform containing thread microfluidics and ISEs on PET substrate	Pot	Pt CE, Ag pseudoRE and ISEs SP with carbon ink; ISEs and REs coated with PEDOT and POT	Sodium/ Potassium	8 min	Na: 52.4 and 56.4 mV/dec for PEDOT and POT resp. K: 45.7 and 54.3 mV/dec for PEDOT and POT resp.	[66]

* Time to respond to changes in concentration. Pot: Potentiometric; Amp: Amperometric; Imp: Impedimetric; WE: Working electrode; RE: Reference electrode; CE: Counter electrode; SP: Screen-printed; PI: Polyimide; PET: Polyethylene terephthalate; PDMS: Polydimethylsiloxane; LOD: Limit of detection; GOx: Glucose Oxidase; IrOx: iridium oxide; PU: polyurethane; BSA: bovine serum albumin; SPEES/PES: sulphonated polyesther ether sulphone—polyether sulphone; LOx: Lactate Oxidase; PEDOT:PSS: poly(3,4-ethylenedioxythiophene) polystyrene sulfonate; PEDOT: poly(3,4-ethylenedioxythiophene); PVB: Polyvinyl butyral; POT: poly(3-octylthiophene-2,5-diyl).
